# Association of Chemoradiotherapy With Thoracic Vertebral Fractures in Patients With Esophageal Cancer

**DOI:** 10.1001/jamanetworkopen.2020.13952

**Published:** 2020-09-01

**Authors:** Kota Fujii, Katsuyuki Sakanaka, Ryuji Uozumi, Yuichi Ishida, Hiroyuki Inoo, Shigeru Tsunoda, Shin’ich Miyamoto, Manabu Muto, Takashi Mizowaki

**Affiliations:** 1 Department of Radiation Oncology and Image-Applied Therapy, Kyoto University Graduate School of Medicine, Kyoto, Japan; 2 Department of Biomedical Statistics and Bioinformatics, Kyoto University Graduate School of Medicine, Kyoto, Japan; 3 Department of Surgery, Kyoto University Graduate School of Medicine, Kyoto, Japan; 4 Department of Gastroenterology and Hepatology, Kyoto University Graduate School of Medicine, Kyoto, Japan; 5 now affiliated with Department of Gastroenterology, National Hospital Organization Kyoto Medical Center, Kyoto, Japan; 6 Department of Therapeutic Oncology, Kyoto University Graduate School of Medicine, Kyoto, Japan

## Abstract

**Importance:**

The association of chemoradiotherapy (CRT) with a thoracic vertebral fracture in patients with esophageal cancer is unknown.

**Objective:**

To determine whether CRT is associated with thoracic vertebral fractures in patients with esophageal cancer.

**Design, Setting, and Participants:**

This retrospective cohort study included patients with clinical stages I to III thoracic esophageal cancer who visited the Kyoto University Hospital, Kyoto, Japan, from January 1, 2007, to December 31, 2013. Data were analyzed from April 6, 2018, to June 4, 2020.

**Exposures:**

Chemoradiotherapy (CRT group) or surgery or endoscopic treatment (non-CRT group).

**Main Outcomes and Measures:**

The main outcome of this study was the cumulative incidence rate of thoracic vertebral fractures in 36 months. The incidence rate was calculated taking censoring into account. Possible risk factors, including CRT, were explored in the multivariable analysis. The association of irradiated doses with fractured vertebrae was also evaluated.

**Results:**

A total of 315 patients (119 for the CRT group and 196 for the non-CRT group) were included. The median age of patients was 65 (range, 32-85) years. Fifty-six patients (17.8%) were female and 259 (82.2%) were male. The median observation time was 40.4 (range, 0.7-124.1) months. Thoracic vertebral fractures were observed in 20 patients (16.8%) in the CRT group and 8 patients (4.1%) in the non-CRT group. The 36-month incidence rate of thoracic vertebral fractures was 12.3% (95% CI, 7.0%-19.1%) in the CRT group and 3.5% (95% CI, 1.3%-7.5%) in the non-CRT group (hazard ratio [HR], 3.41 [95% CI, 1.50-7.73]; *P* = .003). The multivariable analysis showed that the HR of the thoracic vertebral fracture in the CRT group to non-CRT group was 3.91 (95% CI, 1.66-9.23; *P* = .002) with adjusting for sex, 3.14 (95% CI, 1.37-7.19; *P* = .007) with adjusting for age, and 3.10 (95% CI, 1.33-7.24; *P* = .009) with adjusting for the history of vertebral or hip fractures. The HR of the thoracic vertebral fracture for a 5-Gy increase in the mean radiation dose to the single vertebra was 1.19 (95% CI, 1.04-1.36; *P* = .009).

**Conclusions and Relevance:**

This study found that chemoradiotherapy was associated with thoracic vertebral fractures in patients with esophageal cancers. A reduced radiation dose to thoracic vertebrae may decrease the incidence of fractures.

## Key Points

**Question:**

Is chemoradiotherapy associated with thoracic vertebral fractures in patients with esophageal cancer?

**Findings:**

Chemoradiotherapy was associated with thoracic vertebral fractures in a retrospective cohort study of 315 patients with stages I to III esophageal cancer who underwent endoscopic treatment, surgery, or chemoradiotherapy. The irradiated dose applied to a thoracic vertebra was associated with its fracture.

**Meaning:**

Decreasing irradiated doses to the thoracic vertebrae may reduce the incidence of vertebral fractures in patients with esophageal cancer after chemoradiotherapy.

## Teaser

This cohort study assesses whether chemoradiotherapy is associated with thoracic vertebral fractures in patients with esophageal cancer.

## Introduction

Vertebral fractures are the most common osteoporotic fractures in adults.^1,2,3,4^ The incidence rates of a vertebral fracture for European patients older than 55 years were 5.2 to 9.3 per 1000 person-years in men and 7.8 to 19.6 per 1000 person-years in women.^3^ Those for Japanese patients aged 60 to 79 years were 6.5 to 12.8 per 1000 person-years for men and 12.4 to 24.5 per 1000 person-years for women.^5^ Age, sex, body mass index, history of osteoporotic fractures, history of smoking, use of oral glucocorticoids, alcohol intake, history of hip fractures in parents,^6^ and low Hounsfield unit (HU) values^7,8^ were considered risk factors for vertebral fractures. A vertebral fracture decreases a patient’s activity and quality of life^9^ and increases mortality.^10^

Radiotherapy for malignant neoplasms was reported to be associated with vertebral fractures.^11^ The 1- or 2-year cumulative incidence of vertebral fractures was reported as 8% to 19% after preoperative chemoradiotherapy (CRT) for pancreatic cancer,^12^ radiotherapy for locally advanced non–small cell lung cancer,^13^ and stereotactic body radiotherapy for a spinal tumor.^14^ Those incidence rates were higher than those for general osteoporotic fractures.^3,5^ The association of radiotherapy with vertebral fractures was estimated; however, 2 critical flaws existed in those reports that demonstrate the association of radiotherapy with vertebral fractures. First, those reports were based on a study that measured the incidence rate in only those who underwent radiotherapy. No control group was set in their reports.^12,13,14^ Their reports failed to undermine the influences derived from confounding factors such as malignant neoplasm, which is a risk factor for vertebral fractures.^15,16,17^ Second, previous reports did not consider the predilection for vertebral fractures among thoracic vertebrae. Thoracic vertebral fractures frequently arise in the 7th, 8th, 11th, or 12th vertebra.^3,18,19^ Previous reports did not analyze the association of irradiation fields or irradiated doses with the location of a vertebral fracture.^7,8^ Appropriate targets and methods are necessary to demonstrate the association of radiotherapy with vertebral fractures.

Esophageal squamous cell carcinoma is useful for appropriately evaluating the association of radiotherapy with thoracic vertebral fractures. Unlike an adenocarcinoma in a gastroesophageal junction,^20^ a squamous cell carcinoma arises from the thoracic esophagus.^21^ Its treatment is multimodal, consisting of endoscopic treatment, surgery, and CRT. This feature enables us to compare the incidence of vertebral fractures in patients who underwent radiotherapy. The irradiation fields encompassed the primary tumor and metastatic mediastinal lymph nodes along with the thoracic esophagus. A wide range of thoracic vertebrae was included in the irradiation fields. This second feature enables us to investigate whether an association exists between the irradiated doses to a single vertebra and a vertebral fracture.

The aim of this study was to compare the incidence rate of a vertebral fracture in patients with thoracic esophageal cancer treated by CRT with that of endoscopic treatment or surgery using our institutional database. This study also aimed to examine the association of irradiated vertebral doses with the corresponding fracture.

## Methods

This single-institution retrospective cohort study was conducted in accordance with the Declaration of Helsinki^22^ and approved by the institutional review board of Kyoto University on April 6, 2018, which waived the need for informed consent for use of retrospective data. We retrospectively reviewed our institutional database of patients with esophageal cancer who had visited our hospital cancer center. This study followed the Strengthening the Reporting of Observational Studies in Epidemiology (STROBE) reporting guideline. Data were analyzed from April 6, 2018, to June 4, 2020.

### Inclusion Criteria

**Figure 1.**
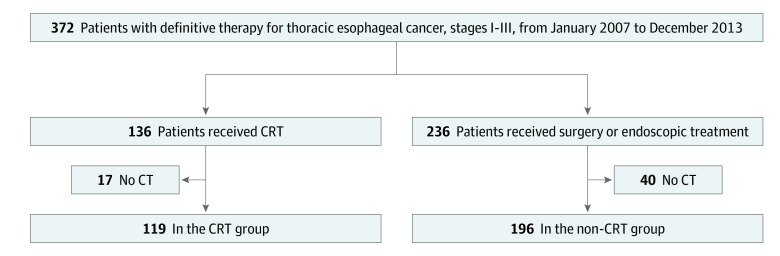
Flow Diagram of Study Population Selection Three hundred and seventy-two patients in our institutional database satisfied the eligibility criteria of this study. Of these, 57 patients were excluded because computed tomographic (CT) scans before the treatments or CT scans at least 90 days after the treatments were not available. Three hundred and fifteen patients were included in this study. CRT indicates chemoradiotherapy.

The inclusion criteria were as follows: (1) a visit to our center, which specialized in the treatment of esophageal cancer, from January 1, 2007, to December 31, 2013, (2) thoracic esophageal cancer, (3) pathologically confirmed squamous cell carcinoma, (4) diagnosis of clinical stages I to III cancer according to the International Union Against Cancer TNM classification,^23^ (5) no history of treatment for esophageal cancer, and (6) receipt of CRT using 3-dimensional conformal radiotherapy, surgery, or endoscopic treatment as a definitive treatment in our hospital. The exclusion criteria consisted of (1) no computed tomographic (CT) scans for all thoracic vertebrae before any treatments and (2) no follow-up CT scans for all thoracic vertebrae at least 90 days after endoscopic treatment, surgery, or the initial day of CRT. According to the inclusion and exclusion criteria, 315 patients were eligible (Figure 1).

### Collected Data

Collected data from medical records included risk factors for a vertebral fracture, such as age, sex, clinical stage of the esophageal cancer, body mass index, history of habitual alcohol use, history of habitual smoking, history of corticosteroid intake, and history of vertebral or hip fractures.^2,4,6,24^ Other collected data consisted of the types of treatment for esophageal cancer, the regimen of concurrent or neoadjuvant chemotherapy, follow-up CT images, the existence of back pain, details of radiotherapy, including the dose distribution to vertebra, and HU values of the vertebrae in the simulation CT for radiotherapy planning.

### Details of Treatments

Computed tomographic simulation was performed in all included patients who underwent CRT. The clinical target volume for the primary tumor was constructed by adding a 0.5-cm cross-sectional margin to the gross tumor volume plus a craniocaudal esophagus with a length of 2 cm each. The clinical target volume for the metastatic lymph node was defined as the gross tumor volume plus a 0.5-cm margin in all directions. The planning target volume was constructed by adding a 0.5-cm margin to the clinical target volume in all directions. Ninety-six patients underwent subclinical lymph nodal irradiation. Subclinical lymph nodes encompassed mediastinal lymph nodes that were defined depending on the location of the primary esophageal tumor.^25^ All radiotherapy plans consisted of multiple (mostly 2 to 4) coplanar beams with 6- or 15-MV energy x-rays. The median prescribed dose for gross tumors was 60 (range, 50-66) Gy and for subclinical lymph nodes was 40 (range, 39-48) Gy in 1.8- to 2.0-Gy fractions. The concurrent chemotherapy regimen was fluorouracil plus cisplatin for 96 patients, fluorouracil plus nedaplatin for 6 patients, fluorouracil only for 14 patients, and intake combination of tegafur, gimeracil, and oteracil for 3 patients.

The non-CRT group included 128 patients who were treated with surgery and 68 patients who were treated endoscopically (n = 196). Of 128 patients who were treated with surgery, neoadjuvant chemotherapy was performed in 83. The neoadjuvant chemotherapy regimen included fluorouracil plus cisplatin for 79 patients, fluorouracil plus nedaplatin for 2 patients, fluorouracil only for 1 patient, and intake tegafur-gimeracil-oteracil plus cisplatin for 1 patient. No patient who received endoscopic treatment underwent chemotherapy.

### Follow-up

Follow-up CTs scanned each patient’s body from the neck to the upper abdomen. The scans were performed approximately every 3 to 4 months until 3 years after treatment and every 6 to 12 months until 4 years after treatment.

### Diagnosis of Vertebral Fractures

Two radiation oncologists (K.F. and K.S.) retrospectively reviewed all CT images of all included patients and confirmed the diagnosis of a thoracic vertebral fracture by consensus. A reconstructed sagittal image and the semiquantitative method of Genant^1,4,5,26^ were used to diagnose vertebral fractures. Vertebral fractures were defined by at least 20% loss in height or 10% loss in areas of vertebral bodies in the sagittal view. Pathological vertebral fractures secondary to bone metastases or direct vertebral invasion of a recurrent tumor were excluded from the event.

### Measurement of Vertebra Doses and HU

We measured the irradiated doses and HU of each thoracic vertebra before CRT in the CRT group. We adopted the HU for quantitative evaluation of baseline osteoporotic change in each thoracic vertebra.^27,28,29,30,31^ Computed tomographic simulation images for radiotherapy were used for calculation. The trabecular bone portion was defined as a region of interest (eFigure in the Supplement) for calculating thoracic vertebral doses and vertebral HU. The mean vertebral dose, the maximum vertebral dose, and the mean HU value in each thoracic vertebra were used for analysis.

### Statistical Analysis

The cumulative incidence rates of a thoracic vertebral fracture in 36 months were calculated in the CRT and non-CRT groups, taking censoring into account. The event of a vertebral fracture was defined as the initial thoracic vertebral fracture after any treatment in all included patients. The follow-up period started from the day of endoscopic treatment, the day of surgery, or the initial day of CRT. The follow-up period ended on the day that the vertebral fracture was initially diagnosed after any treatment, or it was censored on the day of the last follow-up CT in patients without a vertebral fracture. In the case of radiotherapy to the thorax after treatment in patients in the non-CRT group—such as prophylactic radiotherapy after endoscopic treatment, palliative radiotherapy for bone metastases, or radiotherapy for lung cancer—the follow-up period was censored on the initial day of the radiotherapy. The Fine-Gray subdistribution hazards model was used to estimate hazard ratios (HRs) accounting for death as a competing risk with 95% CIs and explore factors associated with thoracic vertebral fractures.^32^ To confirm the association between the risk factors and vertebral fractures, we used the Cox proportional hazards model as a sensitivity analysis for the Fine-Gray model. The variables for the multivariable analyses were selected following the univariable analysis results and the Greenland model-building strategy, which adopted the threshold of a 10% change in the estimate of effect for the main factor.^33^ In case of highly correlated factors, only one of them was chosen as the representative for the multivariable analyses.

A multivariable shared gamma frailty model^34^ with patient-specific random effect was used to examine the association of a vertebral dose, HU, or vertebral location with a vertebral fracture in the CRT group. A total of 1428 vertebrae (12 vertebrae in each of the 119 patients) were independently followed up. Time was calculated from the initial day of CRT. The observation was censored on the day of the last follow-up CT.

All statistical analyses were performed using EZR, version 1.40 (Saitama Medical Center, Jichi Medical University),^35^ a modified version of R commander (R Project for Statistical Computing), and SAS, version 9.4 (SAS Institute Inc). *P* values are reported as continuous quantities.

## Results

**Figure 2.**
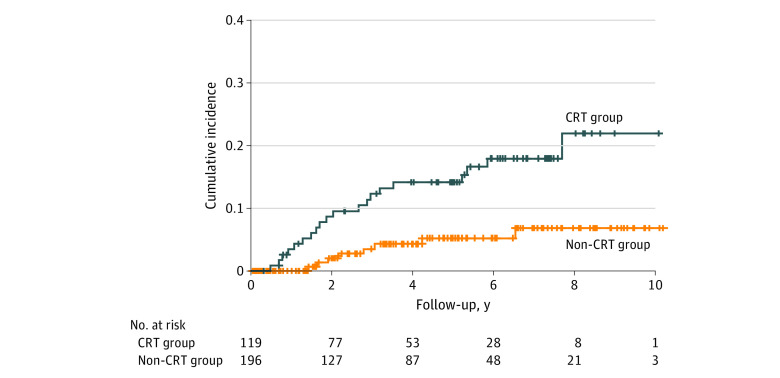
The Cumulative Incidence Rate of Thoracic Vertebral Fractures The cumulative incidence rate of a thoracic vertebral fracture in 36 months in the chemoradiotherapy (CRT) group was 12.3% (95% CI, 7.0%-19.1%). Compared with the non-CRT group (3.5% [95% CI, 1.3%-7.5%]), the CRT group demonstrated the higher cumulative incidence rate of a thoracic vertebral fracture.

Among the 315 patients included in the analysis, the median age was 65 (range, 32-85) years; 56 (17.8%) were female; and 259 (82.2%) were male (Table 1). The median observation time was 40.4 (range, 0.7-124.1) months overall, with 40.3 (range, 4.0-122.7) months in the CRT group and 40.6 (range, 0.7-124.1) months in the non-CRT group. The thoracic vertebral fractures were observed in 20 patients (16.8%) in the CRT group and 8 patients (4.1%) in the non-CRT group. The 36-month incidence rate of thoracic vertebral fractures was 12.3% (95% CI, 7.0%-19.1%) in the CRT group and 3.5% (95% CI, 1.3%-7.5%) in the non-CRT group (Figure 2). The univariable analysis showed that CRT (HR, 3.41 [95% CI, 1.50-7.73]; *P* = .003), being 65 years or older (HR, 3.23 [95% CI, 1.31-7.97]; *P* = .01), being female (HR, 2.83 [95% CI, 1.30-6.13]; *P* = .009), and previous vertebral or hip fractures (HR, 4.63 [95% CI, 1.98-10.90]; *P* < .001) were associated with the development of a thoracic vertebral fracture (Table 2). A history of habitual alcohol use was inversely associated with the development of thoracic vertebral fractures (HR, 0.29 [95% CI, 0.12-0.68], *P* = .005). A history of habitual alcohol use was associated with sex (χ^2^ = 49.59; *P* < .001). To account for this multicollinearity, a history of habitual alcohol use was not included in the multivariable analysis. All 3 patients with a history of corticosteroid intake were censored cases. The multivariable analysis showed that the HR in CRT was 3.14 (95% CI, 1.37-7.19; *P* = .007) with adjusting for age, 3.91 (95% CI, 1.66-9.23; *P* = .002) with adjusting for sex, and 3.10 (95% CI, 1.33-7.24; *P* = .009) with adjusting for the history of vertebral or hip fractures (Table 2). The change in the HR in CRT was higher than 10% when adjusted by sex. The change in the HR in CRT was highest when adjusted by sex also in the sensitivity analyses (range of HRs, 4.54 [95% CI, 1.59-13.00] to 5.58 [95% CI, 1.98-15.70]) (eTables 1-4 in the Supplement).

**Table 1.** Patient Characteristics

Characteristic	Treatment group^a^		
	CRT (n = 119)	Non-CRT (n = 196)	Total (N = 315)
Age, median (range), y	66 (47-84)	65 (32-85)	65 (32-85)
Female	18 (15.1)	38 (19.4)	56 (17.8)
Clinical stage^b^			
I	35 (29.4)	90 (45.9)	125 (39.7)
II	23 (19.3)	70 (35.7)	93 (29.5)
III	61 (51.3)	36 (18.4)	97 (30.8)
BMI, median (range)	20.7 (12.6-31.3)	21.8 (15.1-31.9)	21.4 (12.6-31.9)
History of habitual alcohol use	106 (89.1)	176 (89.8)	282 (89.5)
History of habitual smoking	101 (84.9)	162 (82.7)	263 (83.5)
History of corticosteroid intake	0	3 (1.5)	3 (1.0)
History of vertebral or hip fractures	11 (9.2)	12 (6.1)	23 (7.3)

Abbreviations: BMI, body mass index (calculated as weight in kilograms divided by square of height in meters); CRT, chemoradiotherapy.

^a^ Unless otherwise indicated, data are expressed as number (percentage) of patients.

^b^ Defined using the International Union Against Cancer TNM stage.^23^

**Table 2.** Univariable and Multivariable Analyses Using the Fine-Gray Subdistribution Hazards Model

Variable	No. of patients (n = 315)	No. of events (%)	Univariable		Multivariable (CRT plus univariable)					
			HR (95% CI)	*P* value	HR (95% CI)^a^	*P* value	HR (95% CI)^b^	*P* value	HR (95% CI)^c^	*P* value
CRT										
Yes	119	20 (16.8)	3.41 (1.50-7.73)	.003	3.14 (1.37-7.19)	.007	3.91 (1.66-9.23)	.002	3.10 (1.33-7.24)	.009
No	196	8 (4.1)	1 [Reference]	NA	1 [Reference]	NA	1 [Reference]	NA	1 [Reference]	NA
Age, y										
≥65	172	22 (12.8)	3.23 (1.31-7.97)	.01	2.94 (1.18-7.35)	.02	NA	NA	NA	NA
<65	143	6 (4.2)	1 [Reference]	NA	1 [Reference]	NA	NA	NA	NA	NA
Sex										
Female	56	10 (17.9)	2.83 (1.30-6.13)	.009	NA	NA	3.44 (1.55-7.61)	.002	NA	NA
Male	259	18 (6.9)	1 [Reference]	NA	NA	NA	1 [Reference]	NA	NA	NA
Clinical stage^d^										
III	97	9 (9.3)	1.20 (0.49-2.93)	.68	NA	NA	NA	NA	NA	NA
II	93	9 (9.7)	1.19 (0.49-2.93)	.70	NA	NA	NA	NA	NA	NA
I	125	10 (8.0)	1 [Reference]	NA	NA	NA	NA	NA	NA	NA
BMI										
≤21.4	158	15 (9.5)	1.14 (0.55-2.39)	.73	NA	NA	NA	NA	NA	NA
>21.4	157	13 (8.3)	1 [Reference]	NA	NA	NA	NA	NA	NA	NA
History of habitual alcohol use										
Yes	282	21 (7.4)	0.29 (0.12-0.68)	.005	NA	NA	NA	NA	NA	NA
No	33	7 (21.2)	1 [Reference]	NA	NA	NA	NA	NA	NA	NA
History of habitual smoking										
Yes	263	21 (8.0)	0.52 (0.22-1.23)	.13	NA	NA	NA	NA	NA	NA
No	52	7 (13.5)	1 [Reference]	NA	NA	NA	NA	NA	NA	NA
History of vertebral or hip fractures										
Yes	23	7 (30.4)	4.63 (1.98-10.90)	<.001	NA	NA	NA	NA	3.96 (1.61-9.73)	.003
No	292	21 (7.2)	1 [Reference]	NA	NA	NA	NA	NA	NA	NA

Abbreviations: BMI, body mass index; CRT, chemoradiotherapy; HR, hazard ratio; NA, not applicable.

^a^ Adjusted for age.

^b^ Adjusted for sex.

^c^ Adjusted for history of vertebral or hip fractures.

^d^ Defined using the International Union Against Cancer TNM stage.^23^

The median observation time of single vertebrae was 48.9 (range, 4.0-122.7) months. The incidence rate of thoracic vertebral fractures was 5 per 1000 vertebra-years, which corresponded to 55 per 1000 person-years when converted. Twenty-six thoracic vertebral fractures were observed in 20 of 119 patients (16.8%) during follow-up. Fifteen patients experienced 1 thoracic vertebral fracture, 4 patients experienced 2 thoracic vertebral fractures, and 1 patient experienced 3 thoracic vertebral fractures. Fractures were located at T4 (n = 1), T5 (n = 2), T6 (n = 2), T7 (n = 3), T8 (n = 6), T9 (n = 4), T10 (n = 3), T11 (n = 1), and T12 (n = 4). Nine of 20 patients (45.0%) experienced back pain accompanied by a thoracic vertebral fracture. The median value of a mean thoracic vertebral dose was 37.5 (quantile, 22.4-45.0; range, 0.0-58.9) Gy. The median value of the mean HU was 182 (quantile, 142-213; range, 51-399).

The shared frailty model demonstrated that the incidence of a thoracic vertebral fracture increased depending on the vertebral dose and HU. The HR of a thoracic vertebral fracture after CRT was 1.19 (95% CI, 1.04-1.36; *P* = .009) in a 5-Gy increase of mean thoracic vertebral dose, 0.88 (95% CI, 0.84-0.93; *P* < .001) in a 5-U increase of mean HU, and 3.15 (95% CI, 1.40-7.10; *P* = .006) in women (Table 3). The result of models including the maximum radiation dose and thoracic levels of vertebrae were shown in eTables 5 and 6 in the Supplement; their results were compatible with those above.

**Table 3.** Shared Frailty Model for Single Thoracic Vertebral Fracture

Factor	HR (95% CI)	*P* value
5-Gy increase of mean radiation dose	1.19 (1.04-1.36)	.009
5-U increase of mean HU	0.88 (0.84-0.93)	<.001
Female	3.15 (1.40-7.10)	.006

Abbreviation: HR, hazard ratio; HU, Hounsfield unit.

## Discussion

To our knowledge, this study is the first to investigate the incidence of thoracic vertebral fracture in patients with stages I to III thoracic esophageal carcinoma who underwent endoscopic treatment, surgery, or CRT. The incidence of thoracic vertebral fractures was higher in the CRT group than in the non-CRT group. The irradiated dose to vertebra was associated with a thoracic vertebral fracture.

The present study demonstrated that thoracic vertebral fractures occurred more commonly in patients who underwent CRT compared with those who underwent an endoscopic treatment or surgery. The cumulative incidence rate of vertebral fractures in the CRT group was consistent with those of previous reports of vertebral fractures after radiotherapy.^12,13,14^ The current analysis included known risk factors for vertebral fracture^6^ and identified CRT as an independent risk factor for a thoracic vertebral fracture. The 3-year cumulative incidence rate of a vertebral fracture is 12.3%. This indicates that vertebral fracture after CRT for thoracic esophageal cancer is not rare. Clinicians need to be reminded that a thoracic vertebral fracture is not a rare adverse event after CRT for this population.

The present study showed that the HU of the trabecular bone portion calculated by CT simulation images was associated with a thoracic vertebral fracture. Osteoporosis could have been screened by CT images that were acquired for other purposes than the evaluation of osteoporosis.^27,28,29,30,31^ The present study calculated the HU of the trabecular bone portion on CT simulation images using the treatment planning system. The trabecular portion bears approximately 90% of the vertebral withstand load,^7^ and the bone mineral loss in the trabecular portion is 7 times greater than that of the cortical bones.^36^ The reduction of HU values in the trabecular bone portion reflects a loss of bone mineral density.^27,28,29,30,31^ The present result suggests that CT simulation images and treatment planning systems can be used to calculate the HU of the trabecular bone portion. The baseline vertebral HU on CT simulation images may help to assess the risk of thoracic vertebral fractures in patients who undergo CRT.

A positive correlation was observed between a thoracic vertebral radiation dose and the incidence of a thoracic vertebral fracture in patients treated with CRT. Vertebrae that were frequently fractured secondary to age-related osteoporosis included the 7th, 8th, 11th, and 12th thoracic vertebrae.^3,18,19^ Thoracic vertebral fractures of T4 to T6 were observed in patients treated with CRT in the present study. In these levels, osteoporotic fractures were atypical. The present study suggests that the vertebral dose is possibly associated with vertebral fracture. Proton therapy was reported to reduce doses to vertebrae without compromising target coverage in the radiotherapy planning study.^37^ The incidence of thoracic vertebral fracture after CRT for esophageal cancer may decrease if the vertebral dose is reduced using the advanced radiotherapy techniques.

The pathophysiological mechanism of radiotherapy-induced fracture was reported to be similar to that of age-related osteoporosis. It was characterized by the reduction in osteoblast-mediated bone formation, increased bone marrow adiposity, and apoptosis of osteocytes.^38^ These changes cause a deterioration of skeletal architecture, a loss of bone mineral density, and ultimately a vertebral fracture. It is assumed that the aforementioned pathophysiological mechanism occurs depending on the vertebral radiation dose; thus, vertebral fractures develop depending on the vertebral radiation dose. This pathophysiological mechanism supports the present findings that radiotherapy is associated with vertebral fracture. To reduce the risk of vertebral fractures, the treatment for osteoporosis such as oral supplementation of vitamin D or calcium may be indicated to this population. The automatic contouring or identifying system for the vertebral bodies with low HU and the radiotherapy planning to reduce radiation dose to these vertebrae will be the future work.

### Limitations

Limitations of this study included it being a single-institution retrospective study with a small sample size and a small number of events. The information of some known risk factors, such as history of hip fractures in the parents of patients, were lacking. The differences of patients’ backgrounds existed between the CRT and the non-CRT groups. The effects of radiotherapy or chemotherapy were not individually estimated owing to small number of events in the non-CRT group (eTable 7 in the Supplement).

## Conclusions

In this cohort study, CRT was associated with thoracic vertebral fractures in patients with thoracic esophageal cancers. The HU value and the radiation dose to the vertebra were associated with the occurrence of fractures. A reduced radiation dose to the thoracic vertebrae in chemoradiotherapy may decrease the risk of vertebral fractures.

## Notes

Supplement. **eFigure.** The Delineation of Trabecular Bone Portion (Axial and Sagittal View) **eTable 1.** Univariable and Multivariable Analyses Using the Fine and Gray Subdistribution Hazards Model With Observation Truncated at 3 Years **eTable 2.** Univariable and Multivariable Analyses Using the Cox Proportional Hazards Model **eTable 3.** Univariable and Multivariable Analyses Using the Cox Proportional Hazards Model With Observation Truncated at 3 Years **eTable 4.** Hazards Model With Chemoradiotherapy Plus Clinical Stages **eTable 5.** Shared Frailty Model for Single Thoracic Vertebral Fracture Including Maximum Radiation Dose **eTable 6.** Shared Frailty Model for Single Thoracic Vertebral Fracture Including Thoracic Levels of the Vertebra **eTable 7.** Fine and Gray Subdistribution Hazards Model to Evaluate the Effect of Chemotherapy on Thoracic Vertebral Fractures in Non-CRT Group (n = 196)
