# Supplementary material for: Association of Chemoradiotherapy With Thoracic Vertebral Fractures in Patients With Esophageal Cancer
Source: JAMA Netw Open. 2020 Sep 1;3(9):e2013952. doi: 10.1001/jamanetworkopen.2020.13952 (PMC7489848; doi:10.1001/jamanetworkopen.2020.13952)
Supplement: Supplement. — eFigure. The Delineation of Trabecular Bone Portion (Axial and Sagittal View) eTable 1. Univariable and Multivariable Analyses Using the Fine and Gray Subdistribution Hazards Model With Observation Truncated at 3 Years eTable 2. Univariable and Multivariable Analyses Using the Cox Proportional Hazards Model eTable 3. Univariable and Multivariable Analyses Using the Cox Proportional Hazards Model With Observation Truncated at 3 Years eTable 4. Hazards Model With Chemoradiotherapy Plus Clinical Stages eTable 5. Shared Frailty Model for Single Thoracic Vertebral Fracture Including Maximum Radiation Dose eTable 6. Shared Frailty Model for Single Thoracic Vertebral Fracture Including Thoracic Levels of the Vertebra eTable 7. Fine and Gray Subdistribution Hazards Model to Evaluate the Effect of Chemotherapy on Thoracic Vertebral Fractures in Non-CRT Group (n = 196) [file jamanetwopen-e2013952-s001.pdf]

Supplementary Online Content

Fujii K, Sakanaka K, Uozumi R, et al. Association of chemoradiotherapy with thoracic vertebral fractures in patients with esophageal cancer. *JAMA Netw Open*. 2020;3(9):e2013952. doi:10.1001/jamanetworkopen.2020.13952

eFigure. The Delineation of Trabecular Bone Portion (Axial and Sagittal View)

eTable 1. Univariable and Multivariable Analyses Using the Fine and Gray Subdistribution Hazards Model With Observation Truncated at 3 Years

eTable 2. Univariable and Multivariable Analyses Using the Cox Proportional Hazards Model

eTable 3. Univariable and Multivariable Analyses Using the Cox Proportional Hazards Model With Observation Truncated at 3 Years

eTable 4. Hazards Model With Chemoradiotherapy Plus Clinical Stages

eTable 5. Shared Frailty Model for Single Thoracic Vertebral Fracture Including Maximum Radiation Dose

eTable 6. Shared Frailty Model for Single Thoracic Vertebral Fracture Including Thoracic Levels of the Vertebra

eTable 7. Fine and Gray Subdistribution Hazards Model to Evaluate the Effect of Chemotherapy on Thoracic Vertebral Fractures in Non-CRT Group (n = 196)

This supplementary material has been provided by the authors to give readers additional information about their work.

©2020 Fujii K et al. *JAMA Network Open*.

eFigure. The Delineation of Trabecular Bone Portion (Axial and Sagittal View)

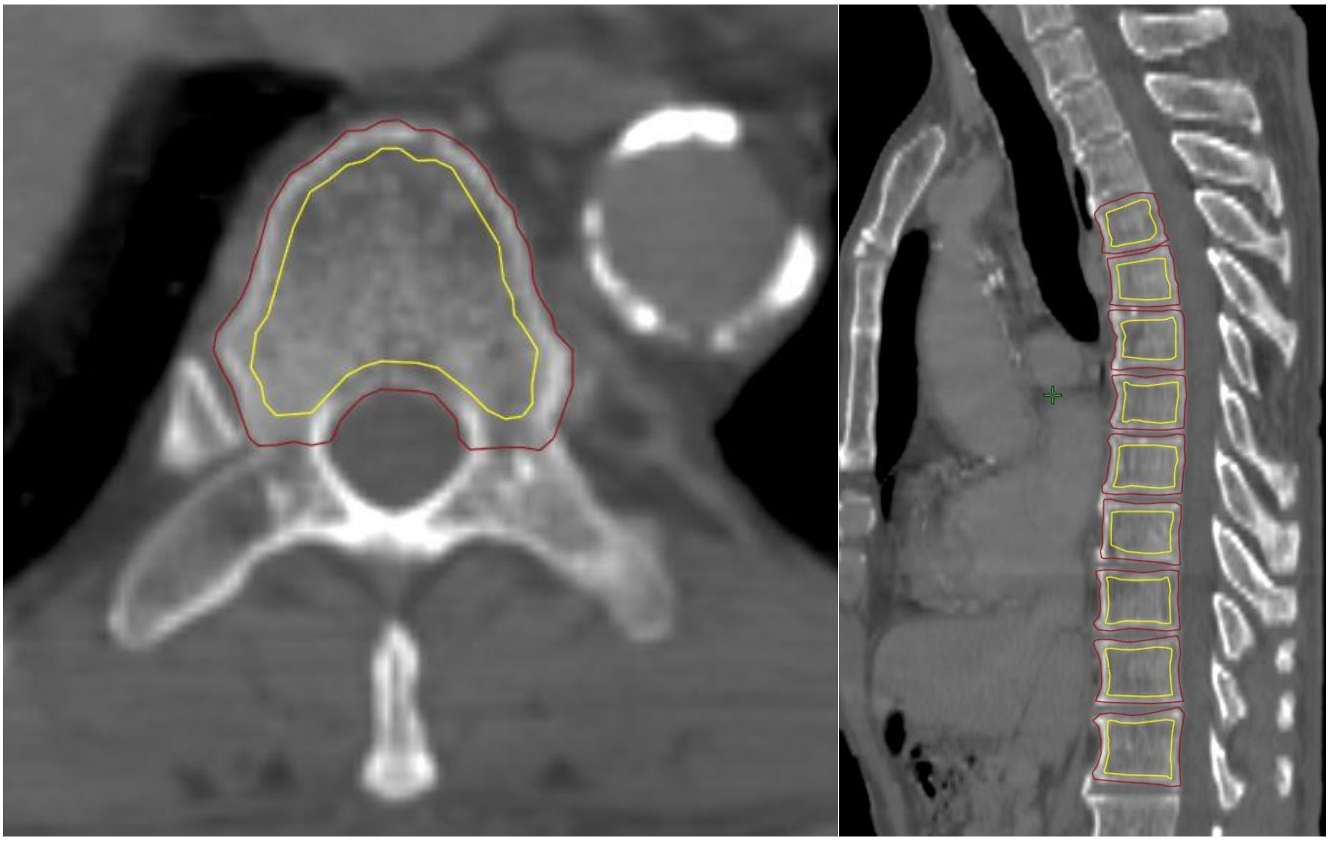

Figure legends: The brown contours meant vertebral bodies. The yellow contours meant the trabecular bone portions. The trabecular portions were created by cropping 3 mm below the surface of the brown contours. Eclipse treatment planning system version 13.7 (Varian Medical Systems, Palo Alto, USA) was used for the analysis.

©2020 Fujii K et al. *JAMA Network Open*.

eTable 1. Univariable and Multivariable Analyses Using the Fine and Gray Subdistribution Hazards Model With Observation Truncated at 3 Years

		Number of patients	Number of events	Univariable		Multivariable (Chemoradiotherapy plus univariable)					
				HR (95% CI)	p value	HR (95% CI)	p value	HR (95% CI)	p value	HR (95% CI)	p value
**Chemoradiotherapy**	Yes	119	14 (11.8%)	3.91 (1.41–10.9)	0.009	3.59 (1.28–10.1)	0.02	4.54 (1.59–13.0)	0.005	3.46 (1.20–10.0)	0.02
	No	196	5 (2.6%)	1		1		1		1	
**Age, years**	≥ 65	172	16 (9.3%)	4.56 (1.31–15.8)	0.02	4.16 (1.17–14.8)	0.03				
	< 65	143	3 (2.1%)	1		1					
**Gender**	Female	56	8 (14.3%)	6.14 (2.38–15.8)	< 0.001			4.25 (1.67–10.8)	0.002		
	Male	259	11 (4.2%)	1				1			
**Clinical Stage (Union for International Cancer Control 6th edition)**	III	97	7 (7.2%)	2.35 (0.70–7.93)	0.17						
	II	93	8 (8.6%)	2.69 (0.82–8.86)	0.10						
	I	125	4 (3.2%)	1							
**Body mass index, kg / m^2**	≤ 21.4	158	12 (7.6%)	1.69 (0.67–4.29)	0.27						
	> 21.4	157	7 (4.5%)	1							
**History of habitual drinking**	Yes	282	13 (4.6%)	0.24 (0.09–0.63)	0.004						
	No	33	6 (18.2%)	1							
**History of habitual smoking**	Yes	263	13 (4.9%)	0.40 (0.15–1.06)	0.07						
	No	52	6 (11.5%)	1							
**History of vertebral or hip fractures**	Yes	23	6 (26.1%)	6.14 (2.38–15.8)	< 0.001					5.22 (1.87–14.6)	0.002
	No	292	13 (4.5%)	1						1	

Abbreviations: HR = hazard ratio; CI = confidence interval.

©2020 Fujii K et al. *JAMA Network Open*.

eTable 2. Univariable and Multivariable Analyses Using the Cox Proportional Hazards Model

		Number of patients	Number of events	Univariable		Multivariable (Chemoradiotherapy plus univariable)					
				HR (95% CI)	p value	HR (95% CI)	p value	HR (95% CI)	p value	HR (95% CI)	p value
**Chemoradiotherapy**	Yes	119	20 (16.8%)	4.05 (1.78–9.19)	< 0.001	3.73 (1.64–8.49)	0.002	5.34 (2.29–12.5)	< 0.001	3.54 (1.54–8.12)	0.003
	No	196	8 (4.1%)	1		1		1		1	
**Age, years**	≥ 65	172	22 (12.8%)	3.33 (1.35–8.22)	0.009	2.99 (1.21–7.40)	0.02				
	< 65	143	6 (4.2%)	1		1					
**Gender**	Female	56	10 (17.9%)	3.01 (1.38–6.55)	0.005			4.52 (2.01–10.2)	< 0.001		
	Male	259	18 (6.9%)	1				1			
**Clinical Stage (Union for International Cancer Control 6th edition)**	III	97	9 (9.3%)	1.85 (0.75–4.57)	0.18						
	II	93	9 (9.7%)	1.36 (0.55–3.36)	0.50						
	I	125	10 (8.0%)	1							
**Body mass index, kg / m^2**	≤ 21.4	158	15 (9.5%)	1.25 (0.60–2.63)	0.55						
	> 21.4	157	13 (8.3%)	1							
**History of habitual drinking**	Yes	282	21 (7.4%)	0.27 (0.11–0.63)	0.003						
	No	33	7 (21.2%)	1							
**History of habitual smoking**	Yes	263	21 (8.0%)	0.55 (0.23–1.29)	0.17						
	No	52	7 (13.5%)	1							
**History of vertebral or hip fractures**	Yes	23	7 (30.4%)	5.63 (2.36–13.5)	< 0.001					4.36 (1.81–10.5)	0.001
	No	292	21 (7.2%)	1						1	

Abbreviations: HR = hazard ratio; CI = confidence interval.

©2020 Fujii K et al. *JAMA Network Open*.

eTable 3. Univariable and Multivariable Analyses Using the Cox Proportional Hazards Model With Observation Truncated at 3 Years

		Number of patients	Number of events	Univariable		Multivariable (Chemoradiotherapy plus univariable)					
				HR (95% CI)	p value	HR (95% CI)	p value	HR (95% CI)	p value	HR (95% CI)	p value
**Chemoradiotherapy**	Yes	119	14 (11.8%)	4.41 (1.59–12.2)	0.004	4.08 (1.47–11.4)	0.007	5.58 (1.98–15.7)	0.001	3.82 (1.36–10.7)	0.01
	No	196	5 (2.6%)	1		1		1		1	
**Age, years**	≥ 65	172	16 (9.3%)	4.60 (1.34–15.8)	0.02	4.21 (1.23–14.5)	0.02				
	< 65	143	3 (2.1%)	1		1					
**Gender**	Female	56	8 (14.3%)	3.57 (1.44–8.89)	0.006			4.84 (1.92–12.3)	< 0.001		
	Male	259	11 (4.2%)	1				1			
**Clinical Stage (Union for International Cancer Control 6th edition)**	III	97	7 (7.2%)	3.29 (0.96–11.3)	0.06						
	II	93	8 (8.6%)	3.00 (0.90–9.96)	0.07						
	I	125	4 (3.2%)	1							
**Body mass index, kg / m^2**	≤ 21.4	158	12 (7.6%)	1.83 (0.72–4.66)	0.20						
	> 21.4	157	7 (4.5%)	1							
**History of habitual drinking**	Yes	282	13 (4.6%)	0.23 (0.09–0.59)	0.003						
	No	33	6 (18.2%)	1							
**History of habitual smoking**	Yes	263	13 (4.9%)	0.43 (0.16–1.13)	0.09						
	No	52	6 (11.5%)	1							
**History of vertebral or hip fractures**	Yes	23	6 (26.1%)	6.30 (2.39–16.6)	< 0.001					5.11 (1.93–13.6)	0.001
	No	292	13 (4.5%)	1						1	

Abbreviations: HR = hazard ratio; CI = confidence interval.

©2020 Fujii K et al. *JAMA Network Open*.

eTable 4. Hazards Model With Chemoradiotherapy Plus Clinical Stages

			Observation					Observation truncated at 3 years				
		Number of patients	Number of events	Fine and Gray subdistribution hazards model		Cox proportional hazards model		Number of events	Fine and Gray subdistribution hazards model		Cox proportional hazards model	
				HR (95% CI)	p value	HR (95% CI)	p value		HR (95% CI)	p value	HR (95% CI)	p value
**Chemoradiotherapy**	Yes	119	20 (16.8%)	4.00 (1.60–10.0)	0.003	4.14 (1.77–9.69)	0.001	14 (11.8%)	4.62 (1.43–15.0)	0.01	4.65 (1.60–13.5)	0.005
	No	196	8 (4.1%)	1		1		5 (2.6%)	1		1	
**Clinical Stage (Union for International Cancer Control 6th edition)**	III	97	9 (9.3%)	0.76 (0.29–1.98)	0.58	1.30 (0.52–3.26)	0.58	7 (7.2%)	1.46 (0.38–5.53)	0.58	2.18 (0.63–7.63)	0.22
	II	93	9 (9.7%)	1.37 (0.55–3.37)	0.50	1.59 (0.64–3.93)	0.32	8 (8.6%)	3.14 (0.98–9.98)	0.05	3.47 (1.04–11.6)	0.04
	I	125	10 (8.0%)	1		1		4 (3.2%)	1		1	

Abbreviations: HR = hazard ratio; CI = confidence interval.

©2020 Fujii K et al. *JAMA Network Open*.

eTable 5. Shared Frailty Model for Single Thoracic Vertebral Fracture Including Maximum Radiation Dose

Factors	Hazard ratio (95% confidence interval)	p value
**5-Gy increase of maximum radiation dose**	1.14 (1.01–1.28)	0.039
**5-units increase of mean Hounsfield unit**	0.89 (0.84–0.93)	< 0.001
**Female**	3.05 (1.35–6.90)	0.007

eTable 6. Shared Frailty Model for Single Thoracic Vertebral Fracture Including Thoracic Levels of the Vertebra

Factors		Multivariable including mean radiation dose		Multivariable including maximum radiation dose	
		HR (95% CI)	p value	HR (95% CI)	p value
**5-Gy increase of mean radiation dose**		1.42 (1.17–1.74)	0.001		
**5-Gy increase of maximum radiation dose**				1.19 (1.01–1.39)	0.03
**5-units increase of mean Hounsfield unit**		0.88 (0.84–0.93)	< 0.001	0.88 (0.84–0.93)	< 0.001
**Female**		4.41 (1.86–10.4)	0.001	3.18 (1.38–7.36)	0.007
**Thoracic levels of the vertebra**					
	**T4**	1		1	
	**T5**	2.02 (0.18–22.3)	0.57	1.68 (0.15–18.5)	0.67
	**T6**	1.88 (0.17–20.8)	0.61	1.40 (0.13–15.5)	0.78
	**T7**	4.07 (0.41–40.2)	0.23	2.33 (0.24–22.6)	0.47
	**T8**	11.05 (1.26–96.8)	0.03	4.90 (0.59–40.8)	0.14
	**T9**	7.33 (0.77–69.5)	0.08	3.53 (0.39–31.8)	0.26
	**T10**	6.20 (0.60–63.7)	0.13	3.01 (0.31–29.2)	0.34
	**T11**	3.46 (0.18–64.8)	0.41	1.02 (0.06–16.7)	0.99
	**T12**	26.43 (1.91–366)	0.02	5.63 (0.53–59.5)	0.15

Abbreviations: HR = hazard ratio; CI = confidence interval.
Footnote: Interpretation should be careful because of the wide CI constructed from the small number of events in each thoracic levels of vertebra.

©2020 Fujii K et al. *JAMA Network Open*.

eTable 7. Fine and Gray Subdistribution Hazards Model to Evaluate the Effect of Chemotherapy on Thoracic Vertebral Fractures in Non-CRT Group (n = 196)

		Number of patients	Number of events (%)	Hazard ratio (95% confidence interval)	p value
**Chemotherapy**	Yes	83	5 (6.0%)	1.93 (0.46–8.15)	0.37
	No	113	3 (2.7%)	1	

Footnote: Interpretation should be careful because of the limited sample size and number of events in non-chemoradiotherapy group.

©2020 Fujii K et al. *JAMA Network Open*.
